# Occupational Stressors and Workplace Challenges Faced by Seafarers in Lithuania: A Cross-Sectional Study

**DOI:** 10.3390/healthcare13111334

**Published:** 2025-06-03

**Authors:** Birute Strukcinskiene, Arturas Razbadauskas, Rasa Grigoliene, Aistė Jeriomenkaite, Jonas Jurgaitis, Vytenis Punys, Vyte Kontautiene, Asta Beniusiene, Dalia Martisauskiene, Erika Zuperkiene, Maria Papadakaki, Donata Zuperkaite, Agnieszka Genowska

**Affiliations:** 1 Faculty of Health Sciences, Klaipeda University, LT-92294 Klaipeda, Lithuania; rektorius@ku.lt (A.R.); vsk.svmf@ku.lt (A.J.); jonas.jurgaitis@ku.lt (J.J.); vytenis.punys@ku.lt (V.P.); vyte.kontautiene@ku.lt (V.K.); asta.beniusiene@ku.lt (A.B.); dalia.martisauskiene@ku.lt (D.M.); 2 Faculty of Marine Technologies and Natural Sciences, Klaipeda University, LT-92294 Klaipeda, Lithuania; rasa.grigoliene@ku.lt; 3 Faculty of Social Sciences and Humanities, Klaipeda University, LT-92294 Klaipeda, Lithuania; erika.zuperkiene@ku.lt; 4 School of Health Sciences, Hellenic Mediterranean University, GR-71410 Heraklion, Greece; mpapadakaki@hmu.gr; 5 Faculty of Medicine, Vilnius University, LT-03101 Vilnius, Lithuania; donata.zuperkaite@mf.stud.vu.lt; 6 Department of Public Health, Medical University of Bialystok, 15-295 Bialystok, Poland

**Keywords:** stress at work, seafarers, occupational stress, work environment, occupational health

## Abstract

Background/Objectives: Seafarers are a particularly isolated workforce, as they are on-site only with their colleagues, both on and off duty. Long-term duties in the sea environment, irregular working hours, changing time zones, and lack of sleep are some of the many factors that negatively affect the physical and mental health of seafarers. This study aimed to explore the occupational stressors faced by seafarers in Lithuania using the HSE Management Standards Indicator Tool (HSE-MSIT). Methods: In 2023, a quantitative study, using a paper survey, of seafarers’ occupational stressors was conducted in Klaipeda city, Lithuania. A total of 385 Lithuanian seafarers participated in the study. Results: The greatest sources of stress at work were identified as *changes at work*, *relationships*, *peer support*, and *management support*. Role clarity, demands, and work control were not strong stressors for the seafarers. Analysis of perceived stress levels revealed notable differences between groups with different work experience and job positions. Analysis of years of service (0–11 years vs. 12+ years) found that seafarers with shorter service experienced more stress in terms of *demands* (*p* = 0.005), *role clarity* (*p* = 0.004), *work control* (*p* = 0.035), and *relationships* (*p* = 0.02). Based on job position (senior vs. junior), junior seafarers experienced significantly higher stress in the *demands* (*p* = 0.001) and *role clarity* (*p* = 0.009) subscales. The study revealed that job position and years of work had weak negative correlations with stress indicators at work. The *Demands* subscale was moderately positively correlated with *relationships* and *change at work*. The *Relationships* subscale was moderately positively correlated with *peer support* and *change at work*. *Role clarity* was moderately negatively correlated with *change at work* and *relationships*. Conclusions: Overall, *changes at work*, *relationships*, *peer support*, and *management support* in the working environment emerged as key factors of perceived stress among seafarers. Junior seafarers and seafarers with shorter years of service experienced higher stress at work. These findings may help in the development of targeted stress management and training strategies tailored to different profiles of seafarers.

## 1. Introduction

The work performed by seafarers is one of the most challenging because seafarers experience unique stressors in their work environment. Representatives of any maritime profession are exposed to unique stressors, such as prolonged stays at sea, separation from family and social life, irregular working hours, crossing multiple time zones, sleep deprivation, extreme temperatures, noise, vibration, strong emotional isolation, and loneliness. Shift work disrupts the natural circadian rhythm and causes chronic fatigue, and the limited living space on board can lead to tension and interpersonal conflicts among the crew. Working under difficult weather conditions and with the risk of accidents, breakdowns, and maritime disasters forces the seafarers to constantly be alert, leading to feelings of strain and stress. All these stressors affect both the physical and mental health of seafarers [1,2,3,4,5,6,7,8,9]. Such factors can lead to increased levels of occupational stress, burnout, and reduced job satisfaction, potentially affecting work efficiency, performance, and safety [2].

Stress can be defined as any type of change that causes physical, emotional, or mental *strain*. Often, this reaction of the body is triggered by unexpected events, which can pose a threat to well-being or cause fear that the situation is poorly controlled. Stress is a set of protective physiological reactions that occur in the human body as a response to the impact of adverse factors (stressors). Our understanding of stress has undergone considerable changes over the last 50 years, and human understanding of the underlying neurobiology has significantly expanded. Instead of considering the biology of stress to be important only in unusual and threatening conditions, it is nowadays understood to be a continuous, adaptive process of examining the environment and coping with adverse situations [10,11,12]. Excessive, continuous stress leads to serious health problems. Psychosocial work-related stress is a risk factor for chronic diseases, especially cardiovascular health disorders [13,14,15,16,17,18,19,20]. Occupational stress increases the risk of cerebrovascular disease and stroke [13,21,22,23,24,25]. Chronic stress can cause eating disorders [26]. Constant long-term stress affects cognitive functions and is associated with fatigue, affective disorders, anxiety, and even depression [12,27,28,29]. Stress at work has a negative impact on sleep quality and can lead to sleep disorders [30,31,32,33,34]. Occupational stress causes work–family conflicts, harms communication with relatives, leading to conflicts with family members or even separation from the family [27,35,36].

In recent years, especially in the context of the COVID-19 pandemic, scientific interest in the topic of seafarers’ mental health and occupational stress has increased significantly, leading to numerous studies and systematic reviews [2,37,38]. One of the main reviews is that by Brooks and Greenberg, which analyses 63 studies published between 2012 and 2021 and identifies the main risk factors for mental health in seafarers. A number of risk factors were identified, including demographic factors (young age, singleness, low professional status), environmental factors (exposure to noise and vibration, extreme weather conditions), organizational factors (long working hours, irregular shifts, lack of autonomy, uncertainty of work schedules), psychosocial factors (isolation, lack of social support, team conflict), and individual factors (low mental resilience, previous health problems). The authors recommend closer monitoring of crew mental health, education, and support for workers [2]. Another important review by Jonglertmontree et al. aimed to identify mental health problems and associated factors among seafarers. After analyzing 24 studies, the authors found that the most common problems were stress, depressive symptoms, and burnout. Risk factors were divided into individual (professional experience, age, health status, mental resilience) and environmental (time pressure, long working hours, noise, vibration, lack of support). Effort–reward imbalance was also identified as a significant factor affecting mental health. The authors emphasize the need for interventions at both the individual level (promoting healthy habits, developing mental resilience) and the organizational level (providing instrumental support, working time management) [37]. Senbursa conducted a literature review on seafarers’ well-being on board. After analyzing 64 articles, of which 14 met the inclusion criteria, the author identified factors affecting seafarers’ mental and physical health, such as depression, anxiety, isolation, insomnia, malnutrition, and fatigue. The author emphasizes the need to promote an organizational culture that supports seafarers’ mental and physical health [38]. These reviews demonstrate the growing recognition of the importance of seafarers’ mental health and the need for further research in this area.

One of the comprehensive options for analyzing occupational stress experienced by seafarers is the Job Demands–Resources Model (JD–R) developed by Demerouti, Bakker et al., which describes a widely used theory of occupational stress [39]. The model assumes that each job has its own demands and resources, and their relationship influences the level of stress, engagement, and burnout of the worker. Demands are the physical, mental, social, or organizational aspects of work that require an employee’s effort and are associated with certain psychophysical costs. On the other hand, resources are elements of work that help achieve professional goals, reduce demands and related costs, and promote the employee’s personal and professional development. When adequate resources do not compensate for these needs, serious psychological consequences can result, including chronic stress, reduced motivation, and even burnout [39]. The JD-R model is based on two main psychological processes: the health impairment process and the motivation process. In the first case, excessive demands at work exhaust the employee’s energy resources, leading to burnout, health problems, and reduced work efficiency. The second process suggests that appropriate job resources can increase employee engagement, leading to better work performance, greater job satisfaction, and a lower likelihood of leaving the job. An important extension of the JD-R model is the inclusion of personal resources, such as self-efficacy, optimism, and mental resilience. These resources play a key role in coping with work demands and can act as a buffer against the negative effects of stress. Employees with high personal resources are more resistant to stress, more committed to work, and achieve better professional results [9,39,40]. The ability to communicate with family, crew rotation, rest, and clearly defined responsibilities are especially effective for seafarers. In maritime organizations, the implementation of JD-R-based strategies can increase the psychological well-being of the crew and reduce employee turnover. This model not only helps to identify sources of stress but also provides directions for effective actions to maintain seafarers’ health and engagement under high-stress conditions. The application of the JD-R model to the analysis of occupational stress experienced by seafarers allows for the identification of specific areas of intervention, such as improving working conditions, increasing social support, and implementing stress management programs, which can contribute to improving the mental well-being of this occupational group [9,40,41,42]. The JD-R model can be successfully applied in various countries. For example, a study in Italy showed that high job demands, such as workload and cognitive load, are significant factors in burnout and poor health among seafarers. However, professional resources, such as social support and transformational leadership, had a preventive effect on burnout and a direct effect on health improvement [43].

Seafarers’ duties are determined by a hierarchical organizational structure that involves rotating shifts and unpredictable work–rest schedules. The ship’s crew is usually divided into separate departments, including the deck department, the engineering department, and the steward department. Each department has different responsibilities and tasks. For example, the deck department is responsible for navigation and cargo handling, the engineering department is responsible for maintaining the ship and machinery, and the steward department is responsible for providing food and accommodation services. The organizational structure of a maritime vessel acts as a compact, self-governing institution in which each person performs different and important duties. The ship crew’s hierarchical structure establishes a system of authority that should allow seafarers to understand their roles and responsibilities [37,44]. This structure aims to prevent confusion and ensure that each seafarer understands their own duties and obligations within the team. However, the rigidity of such hierarchies can also contribute to stress, especially for lower-ranking personnel, as they may face limited autonomy and high accountability for tasks under pressure. Moreover, the ship’s self-contained nature creates a closed social and operational environment. Crew members must rely on each other for both professional collaboration and personal support. This reinforces the need for clear communication, mutual respect, and adherence to protocols within and across departments. Despite its strengths in maintaining order and safety, the hierarchical system can sometimes exacerbate interpersonal tensions and stress, particularly if communication gaps or inequities in workload arise [2]. Inequities in workload distribution have also been reported, where junior seafarers often face disproportionate responsibilities with limited autonomy, contributing to stress and dissatisfaction [35]. Additionally, strict adherence to rank-based authority can hinder the formation of supportive relationships, which are crucial for coping with stress in confined and demanding environments [37,45].

High-risk areas of seafarers’ duties need to be identified and thoroughly analyzed to determine which stressors are dominant in the seafarers’ work environment. This would help in the management of the occupational stress experienced by seafarers and the development of stress-reducing and health-promoting strategies, as well as inform the planning of stress management policies.

To the best of our knowledge, there is limited scientific evidence on the stress experienced by seafarers in the work environment in the European population. For example, a study of Finnish merchant fleet seafarers investigated stress and health among seafarers and revealed that occupational factors, such as noise, climate conditions on board, occupational group/position, and recognition at work, were significant factors associated with stress and health [46]. A study of Polish seafarers analyzed the occurrence of harmful psychosocial factors at work and their stress-coping strategies. The results showed a significant impact of traumatic experiences on the mental and physical health of seafarers [47]. An international study involving European countries revealed the impact of environmental, social, and health factors on the mental health of seafarers and their decision to continue working in the maritime sector [48].

To date, no study has been conducted to assess the stressors affecting seafarers in the Baltic States. However, identifying factors that affect seafarers’ health and well-being is of interest in a coastal country such as Lithuania, a particular geographical area by the Baltic Sea, and a relevant place for exploring seafarers’ issues. Klaipeda city is the only place in Lithuania where seafarers reside, and their health can be studied.

Klaipeda University is a university located in the Lithuanian port city of Klaipeda, a member of the EU-CONEXUS, an alliance of universities from the coastal cities in Europe, incorporating the Faculty of Health Sciences, the Faculty of Marine Technologies and Life Sciences, and the Marine Research Institute. Klaipeda University has adequate facilities to conduct research on seafarers’ health and well-being, and the University researchers are concerned about the health and safety of local and regional seafarer communities. Similar studies could be conducted in other EU-CONEXUS alliance members in the future, and the obtained trends analyzed.

This study addresses the following research question: *What are the main occupational stressors affecting Lithuanian seafarers?* The novelty of the study lies in its innovative approach, using the HSE Management Standards Indicator Tool (HSE-MSIT) to explore the sources of work-related stress among Lithuanian seafarers, with particular attention on differences in job roles and levels of experience. The originality of the research lies in presenting original data on the stress experienced by Lithuanian seafarers, highlighting differences between junior and senior officers, as well as across varying lengths of service, in an area that, until now, has not been thoroughly explored either nationally or within the broader regional context.

This study aimed to explore the occupational stressors faced by seafarers in Lithuania using the HSE Management Standards Indicator Tool (HSE-MSIT). The findings contribute to a growing understanding of seafarer well-being and the need for comprehensive occupational health approaches.

## 2. Materials and Methods

### 2.1. Study Design and Participants

In 2023, a quantitative study of seafarers’ occupational stress was conducted in Klaipeda city, Lithuania, using a written survey. At the time of the study, approximately 6000 seafarers were registered in Lithuania, which is the entire size of the general population. Applying the Paniott formula with a 5% error gave a sample size of 375 respondents. Participants were randomly enrolled in the study; all seafarers were invited to participate and had an equal chance of being included in the study sample. In addition, all seafarers in the study population attended special training courses organized by the Lithuanian Maritime Academy. To properly reflect the Lithuanian seafarers’ population and ensure generalizability, 400 questionnaires were distributed on-site, and 385 were finally completed (96% response rate).

Participants were informed that their participation was voluntary, that they could refuse to participate or withdraw at any time without giving any reason, that all measurement tools were anonymous, and that only summarized data would be reported. By completing the questionnaire, participants provided consent to participate in the assessment of work-related stress.

### 2.2. Measurement Tool

The Health and Safety Executive (HSE), Britain’s national regulator for workplace health and safety, elaborated the HSE Management Standards Indicator Tool (HSE-MS IT) in 2004. Since then, it has become a well-known and validated tool for assessing and analyzing occupational stressors worldwide. The Lithuanian version of the questionnaire (called “SDV”), which was used in this research, was previously validated by the Lithuanian Institute of Hygiene [49,50]. The HSE-MSIT questionnaire consists of 35 items, divided into seven subscales according to the following defined domains: “demands” (8 questions), “managerial support” (5 questions), “peer support” (4 questions), “relationships” (4 questions), “change at work” (3 questions), “work control” (6 questions), and “role clarity” (5 questions). Respondents’ responses were rated on a five-point Likert scale ranging from 1 (never) to 5 (always) or 1 (strongly disagree) to 5 (strongly agree). The variables of the subscales “demands” and “relationships” are scored in reverse according to the scale key: from 5 (never) to 1 (always) or from 5 (strongly disagree) to 1 (strongly agree). A higher score indicates better management standards and, accordingly, a lower risk of work-related stress [49,51].

### 2.3. Study Variables

The study explored participants’ age, gender, years of service (0–11 years; 12+ years), crew size (1–25 persons; 26+ persons), and job position (senior positions included captains and expert staff such as mechanics, electricians, and engineers; junior positions included sailors, cadets, and service staff).

### 2.4. Statistical Analysis

Statistical analysis was conducted using the SPSS 24.0 software program. For the HSE-MSIT, the overall Cronbach’s Alpha coefficient was calculated to be 0.766. This indicates a sufficiently high internal consistency. The Cronbach’s Alpha coefficients for the subscales were higher (Table 1), indicating even better internal consistency of the subscales.

Derived variables were generated based on the HSE-MSIT scale key and analyzed for normal distribution using the Kolmogorov-Smirnov statistical criterion. It was found that the assumption of normality was violated (Table 2); thus, non-parametric criteria were chosen for statistical analysis. Friedman’s criterion was used to compare the means and mean ranks of the components of each subscale. The Mann–Whitney statistical criterion was applied to assess the statistical differences between the two groups, and the effect size r was calculated.

The effect Size interpretation: 0.1 ≤ |r| < 0.3—small, 0.3 ≤ |r| < 0.5—medium, |r| ≥ 0.5—large effect size.

Spearmen’s correlation was calculated to assess the relationships between grouping variables and HSE-MSIT subscales. A larger absolute value of the coefficient represents a stronger relationship between variables. The strength of the correlation was categorized as follows: negligible (0.00–0.10), weak (0.10–0.39), moderate (0.40–0.69), strong (0.70–0.89), and very strong (0.90–1.00) [52,53]. Statistical significance was set at 0.05.

### 2.5. Ethical Considerations

The study was conducted in accordance with the Declaration of Helsinki [54]. The Bioethics Committee of Klaipeda University approved the study protocol (Ref. No. 46Sv-VS-03).

## 3. Results

In total, 385 seafarers participated in the study (367 male (95.3%); 18 female (4.7%)). The average age of the participants was 36.9 ± 10.7 years. The youngest participant was 19 years old, and the oldest was 64 years old. The median age was 38 years.

Similarly, the age distribution among participants was markedly uneven, which limited the feasibility of subgroup analysis by age. Instead, the study focused on years of service within the maritime sector, as this provided a more consistent and analytically relevant basis for examining patterns of occupational stress.

A total of 205 respondents had 0–11 years of service (53.2%), and 180 respondents had more than 12 years of service (46.8%). Based on their job position, there were 202 (52.5%) seniors and 183 (47.5%) juniors. A total of 176 (45.7%) seafarers worked in small crews (up to 20 crew members), and 209 (54.3%) worked in large crews (21+ members).

**Figure 1:**
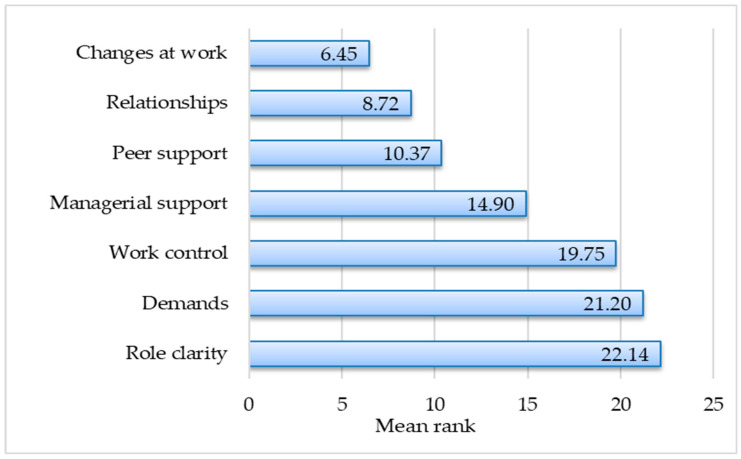
Perceived stress mean ranks within the subscales.

The mean rank differences in the items were compared across all HSE-MSIT subscales, including demands, managerial support, peer support, relationships, change at work, work control, and role clarity (Table 3, Figure 1), based on the Friedman test results. All subscales showed significant differences between item responses (*p* < 0.05), indicating significant variance within groups.

Higher mean ranks indicate that higher response values were obtained in these subscales. This is also confirmed by the analysis of mean values (a 5-point Likert scale was used). We note that the data distribution sequence remains the same; however, in the Friedman test, the mean rank values range from 1.89 to 6.51. Based on the methodology for evaluating the results of the study, higher scores indicate better management standards and therefore a lower risk of work-related stress. The Friedman test reveals the contribution of each variable to the perceived level of stress.

The demands subscale showed the highest level of internal variation (χ^2^ = 720.424, dƒ = 7, *p* < 0.001). It revealed problem areas that did not reach a mean of 3 points, indicating that the most difficult areas to achieve success in are “I have unachievable deadlines” (2.14), “I am pressured to work long hours” (2.37), “I am unable to take sufficient breaks” (2.46), “Different groups at work demand things from me that are hard to combine” (2.51), “I have unrealistic time pressures” (2.52), and “I have to neglect some tasks because I have too much to do” (2.57).

The managerial support subscale (χ^2^ = 643.139, dƒ = 4, *p* < 0.001) indicates the availability of supervisory support. The lowest-rated items were “I can talk to my line manager about something that has upset or annoyed me about work” (2.15), “I am supported through emotionally demanding work” (2.44), and “My line manager encourages me at work” (2.51). This means that seafarers’ relationships with their supervisors are not favorable, and respondents do not feel supported or valued.

The peer support subscale also revealed significant variance (χ^2^ = 608.488, dƒ = 3, *p* < 0.001). The low means for the items “I get help and support I need from colleagues” (1.98), “I receive the respect at work I deserve from my colleagues” (2.05), and “My colleagues are willing to listen to my work-related problems” (2.24) indicate a lack of sufficient emotional support among peers. More than half of the statements revealed that relationships with colleagues are poor.

The dynamics of interpersonal relationships in the relationships subscale revealed a statistically significant difference (χ^2^ = 281.965, dƒ = 3, *p* < 0.001). The lowest mean-rated items were “I am subject to personal harassment in the form of unkind words or behavior” (1.75) and “I am subject to bullying at work” (1.98). The results also revealed that “Relationships at work are strained” (2.52) and “There is friction or anger between colleagues” (2.46) were statistically significant. This is worrying because the averages for the entire subscale are very low, indicating the presence of stress.

The change at work subscale was found to be significant (χ^2^ = 8.076, dƒ = 2, *p* = 0.018). Change at work causes significant stress for seafarers, as indicated by the items “When changes are made at work, I am clear how they will work out in practice” (2.21), “Staff are always consulted about change at work” (2.14), and “I have sufficient opportunities to question managers about change at work” (2.11). These very low averages suggest that supervisors do not consult with seafarers or discuss changes at work with them, creating conditions that contribute to high levels of stress.

The work control subscale (χ^2^ = 570.038, dƒ = 5, *p* < 0.001) provides a range of perceptions of autonomy. Participants reported the lowest mean scores for “I have a say in my own work speed” (2.34) and “My working time can be flexible” (2.77). This means that there is no flexible schedule at work and it is also not possible to choose the pace of work; thus, even if the goals are attainable, the choice of tasks and methods remains limited.

The role clarity subscale (χ^2^ = 127.852, dƒ = 4, *p* < 0.001) confirms the general understanding of particular roles. The highest-rated item was “I am clear what my duties and responsibilities are” (4.59). Participants reported moderate mean scores regarding their supervisor’s expectations (“I am clear what is expected of me at work” (4.53) and “I understand how my work fits into the overall goal of the organization” (4.22)), indicating that the integration of clarity of roles at the organizational level does not act as a stressor.

Perceived stress levels were assessed using the Health and Safety Executive (HSE-MSIT) subscales. Figure 1 shows the differences in perceived stress levels across the subscales. This provides insights into stressors associated with demands, support systems, and the work environment. The highest perceived stress levels were *changes at work* (mean rank 6.45), *relationships* (mean rank 8.72), *peer support* (mean rank 10.37), and *management support* (mean rank 14.90). This indicates that the higher the mean rank, the lower the stress level, and vice versa, the lower the mean rank, the higher the stress level (Figure 1).

The analysis of perceived stress levels revealed notable differences between groups with different work experiences and job positions (Table 4). Using the Mann–Whitney U test, significant differences were identified between the groups in the following subscales:

The following results were obtained within the subscales based on the years of service (0–11 years vs. 12+ years): demands (z = −2.821, *p* = 0.005), small effect size (r = −0.143); managerial support (z = −2.077, *p* = 0.038), small effect size (r = −0.106); relationships (z = −2.333, *p* = 0.02), small effect size (r = −0.119); work control (z = −2.113, *p* = 0.035), small effect size (r = −0.108); role clarity (z = −2.859, *p* = 0.004), small effect size (r = −0.145). Comparing the average ranks by length of service, we observe that seafarers with shorter service duration (0–11 years) experience more stress in the areas of demands (average rank 178.05, *p* = 0.005), relationships (average rank 180.69, *p* = 0.02), work control (average rank 180.39, *p* = 0.035) and role clarity (average rank 177.10, *p* = 0.004), while seafarers with longer service duration (12+ years) experience more stress in the area of managerial support (average rank 180.66, *p* = 0.038).

Based on job position (senior vs. junior), junior seafarers experience more stress in all work-related areas; however, statistically significant differences were found in the demands subscale (mean rank 172.56, z = −3.440, *p* = 0.001), with a small effect size (r = −0.175) and in the role clarity subscale (mean rank 177.51, z = −2.631, *p* = 0.009), with a small effect size (r = −0.134). This indicates that junior workers potentially experience more stress in the demands and role clarity domains.

Spearman’s correlation coefficients were calculated to assess the correlations between variables such as years of service and job position and the seven HSE-MSIT subscales (Table 5). The study identified mostly negligible or weak correlations between job position and all seven subscales, as well as between years of employment and all the subscales. Job position had a slightly negative correlation with years of service (r = −0.277, *p* < 0.001).

The correlations between HSE-MSIT subscales showed moderate positive correlations between *demands* and *relationships* (r = 0.604, *p* < 0.01), as well as between *demands* and *change at work* (r = 0.410, *p* < 0.01), *peer support* and *relationships* (r = 0.433, *p* < 0.01), and *relationships* and *change at work* (r = 0.446, *p* < 0.01).

Negative correlations were found between *change at work* and *role clarity* (r = −0.495, *p* < 0.01), *relationships* and *role clarity* (r = −0.458, *p* < 0.01), and *role clarity* and *peer support* (r = −0.351). Additionally, the *demands* subscale was slightly negatively correlated with *work control* (r = −0.209, *p* < 0.01) and role clarity (r = −0.343, *p* < 0.01). This indicates that increases in *demands* are associated with role ambiguity, and higher demands are associated with reduced feelings of control.

## 4. Discussion

### 4.1. Main Findings

This study offers insights into the stressful experiences of seafarers and the various forms of support that influence their well-being. Participants identified several key stressors, including organizational changes, interpersonal tensions, limited peer and managerial support, and the demanding nature of their roles. While job demands, especially those associated with time pressure and workload, were widely recognized as significant, role clarity presented a more nuanced picture. Although the role clarity subscale showed generally high scores, statistically significant differences (*p* = 0.004) and moderate negative correlations with change at work (r = –0.495) and relationships (r = –0.458) indicate its importance as a stressor for certain groups, particularly junior staff.

While overall ratings for role clarity were positive, this subscale did not show the highest level of agreement, particularly among junior staff. A consistent theme was a lack of autonomy in how tasks were performed. The findings from the work control subscale suggest that many seafarers have minimal influence over the execution of their duties, reflecting a broader limitation in decision-making capacity.

When exploring differences between groups, those with fewer years of service (0–11 years) reported higher stress levels across multiple dimensions: *role clarity*, *job demands*, *work control*, *and relationships*. Junior crew members appeared to be disproportionately affected, with significantly greater stress observed in areas associated with workload and ambiguity in their responsibilities.

Correlation analysis further highlighted subtle but meaningful patterns. Lower-ranked positions were modestly linked to higher perceived stress, and shorter service duration appeared to compound this effect. Interestingly, there were moderate positive links between high job demands and strained interpersonal relationships, as well as frequent organizational changes. Peer support and change at work were also moderately associated with the quality of workplace relationships; conversely, role clarity was moderately and negatively associated with both interpersonal tensions and changes in the work environment, implying that ambiguity about one’s duties may exacerbate stress during periods of transition or conflict.

### 4.2. Analysis of Results

This study provides new evidence for the impact of occupational stressors among Lithuanian seafarers, emphasizing the importance of job demands, autonomy, managerial support, and role clarity. These findings are in line with international research on seafarers’ mental health and workplace challenges [2,5,27].

Although the role clarity subscale gave overall high average scores, some respondents reported uncertainty in their responsibilities, as evidenced by moderate average scores for the items “It is clear what my duties are” (3.39) and “It is clear what my manager expects of me” (3.20). This indicates that role ambiguity, while not universal, is still an issue among certain groups. Hayes-Mejia and Stafström (2024) underlined the importance of clearly defined roles and structured communication in improving ownership and alleviating role ambiguity onboard [5].

Workload-related stress was the most prominent theme, as demonstrated by the mean rank of the statement “I have to work very intensively”, which indicates a significant level of work demands. Ali et al. (2023) similarly highlighted the importance of organizational structure, leadership, and an unambiguous, embodied process of communication to mitigate the negative consequences of high work demands [7]. Stressors beyond workload have also been implicated in psychological distress among maritime personnel as a result of job insecurity, especially when insecurities arise from vague expectations and rigid structures [2].

Another important aspect was the lack of autonomy in decision-making. Participants gave low ratings for items such as “I can control my own work pace” and “I can have flexible work hours,” indicating limited opportunity for seafarers to influence how and when their work is carried out. This supports Jonglertmontree et al. (2022), who noted that limited autonomy and inadequate communication compound mental health difficulties in seafarers [37]. Increasing the ability of seafarers to influence their own work schedule could alleviate some stress and enhance well-being. Ali et al. (2023) noted that rigid work schedules were a major cause of workplace stressors [7]. Practical support was appreciated, but emotional support was perceived as lacking. Seafarers reported valuing aspects of practical support, such as “I can rely on my immediate manager for resolving a work issue” (mean 4.09), but they noted a lack of emotional support. This follows a trend established by Doyle et al. (2016), who found that team dynamics and support from leaders help foster resilience among crew members [45]. By contrast, Dohrmann et al. (2020) found that employee well-being was negatively affected when they did not receive managerial support, particularly when workload was high [27].

It is important to note that although interpersonal tensions were not the primary cause of stress, some respondents reported strained interpersonal relationships with colleagues. These findings highlight the need for appropriate team assessments and training in conflict resolution skills. This is particularly the case for larger or hierarchical crews that are highly susceptible to communication breakdowns.

Variation in stress was also analyzed in relation to variables such as job position and years of service (experience). For example, junior and less experienced seafarers reported demonstrably higher stress in areas such as role clarity, work control, interpersonal relationships, and demands. The findings were similar to those of previous research, which suggested that it was more burdensome psychologically for early-career seafarers who had less experience and therefore often less power [2,45]. Finally, senior crew members indicated that they experienced more stress associated with their lack of managerial support, which may reflect higher expectations and operational responsibility over operational outcomes.

Significantly, while some studies suggest a relationship between higher-ranking positions and resilience, other studies further suggest that command responsibility may contribute to chronic stress. For instance, Jensen and Oldenburg noted that ship officers experience elevated stress levels regardless of age, primarily due to their command responsibilities. They recommend implementing regular training in sleep hygiene, fatigue management, and adherence to rest regulations to reduce long-term risk [7]. In conclusion, this study confirms that high job demands, limited autonomy, and role ambiguity are perceived by seafarers as key occupational stressors. These results reflect the necessity of strengthening organizational clarity and improving leadership communication and participatory environments to safeguard mental health and operational performance at sea.

### 4.3. Stress Management in Seafarers

Various stress management tools and models are offered for stress prevention [37,55,56]. The study highlights the critical areas where improvements can be made to enhance organizational well-being and reduce occupational stress experienced by seafarers. By strengthening managerial supervision, clarifying roles, promoting task autonomy, and addressing working atmosphere issues, maritime organizations can create a more supportive work environment that prioritizes the mental health and well-being of seafarers. Thus, it seems imperative for the maritime industry to implement a comprehensive work organization system that prioritizes both physical and psychological well-being. This system has to promote effective communication, provide mental health services, and offer training opportunities for skill development. Such an approach not only addresses the immediate needs of seafarers but also contributes to their long-term mental health and resilience in the demanding maritime environment.

To ensure physical health, it is recommended that additional regular health check-up programs be implemented. In addition to regular seafarers’ medical examinations and traditional tests to determine whether a person can work, these programs would also include heart disease tests, immune system screening, and general blood tests. Another effective way of helping to ensure better physical health among seafarers is physical therapy during the trip. Physiotherapy would help reduce inflammation and acute and chronic pain, strengthen and relax muscles, improve joint function, speed up the healing of wounds and ulcers, improve blood circulation, digestive tract function, breathing, and other systems, stimulate tissue regeneration processes, and strengthen the body’s immune system. Seafarers would be given the opportunity to attend physiotherapy before and after shifts. Another proposed, equally important aspect in physical health promotion is healthy lifestyle measures. In practice, these could be implemented by offering healthier foods, yoga classes, and physical activity group classes at least once a week.

To strengthen mental health, an additional psychological or mental health check-up should be offered at least once a year. During such check-ups, it would be possible to observe whether and how the psychology of seafarers changes. If certain mental health disorders appear, additional psychological counseling would be offered to the employees, the purpose of which would be to provide timely notice and help seafarers with this type of problem. To strengthen psychological and mental health, seafarers should be offered access to a professional psychologist. Other psychological interventions could also be applied, for example providing informational posters with the contact information (phone number, e-mail) of a person who can provide psychological help during the trip. Communication and therapy measures could also include stress management training for seafarers at least once a year. It is also recommended to organize post-traumatic support group sessions during which seafarers who have experienced the same trauma (ship breakdown during a voyage, piracy, loss of a colleague, etc.) could participate. Another proposed method is the creation of a framework for dealing with difficult situations. Specialists would provide the seafarers with appropriate tools for stress management and explain their mechanisms during group sessions. During such classes, action plans for addressing various problems would be created, and each participant would be helped to identify relaxing activities that distract from the problems and develop changes in mindset when stressful situations arise. Various trainings are recommended at least once every six months. Suicide prevention training, leadership training, anti-bullying, and anti-harassment training are suggested future trainings.

In summary, organizations working with seafarers should consider implementing a comprehensive work system that prioritizes both physical and mental well-being. Such a system should foster effective communication, offer therapy services, and provide access to ongoing professional training. Key elements include regular health screenings, psychological counseling, social engagement activities, and continuous education on stress reduction techniques. In addition, organizations should facilitate communication between seafarers and their families by ensuring access to the internet and telecommunication services while at sea. These measures can help mitigate feelings of isolation and improve overall psychological well-being. Finally, by providing access to recreational activities, nutritious food, and designated training spaces, organizations can promote both health and job satisfaction. A well-designed and supportive work system fosters a positive organizational culture and improves long-term performance and retention in the maritime sector.

### 4.4. Strengths and Limitations

The current study expands our understanding of the workplace stressors experienced by seafarers and provides valuable insights into strategies for reducing stress, enhancing physical health, and promoting mental well-being within this profession. However, the study has some limitations. One significant limitation is the relatively small sample size, which may not fully represent the diverse experiences of seafarers across different types of vessels and maritime environments. By including participants from a wider range of vessels—cargo ships, tankers, and passenger ships—future studies could capture a more comprehensive picture of the occupational stressors encountered in the maritime industry. Additionally, expanding the geographical scope to include seafarers from neighboring maritime countries would enhance the generalizability of the findings. Different cultural contexts and regulatory environments can influence how stressors are perceived and managed, making it crucial to consider these factors in future research. Another limitation is the lack of analysis of gender differences in workplace stressors among seafarers. This omission presents an opportunity for future research to explore how gender dynamics influence stress experiences and coping mechanisms. Previous studies have indicated that male and female seafarers may experience and respond to stress differently due to various social and psychological factors.

An additional notable limitation of this research is its reliance on a cross-sectional approach. This design limits the ability to determine cause-and-effect relationships or observe how variables evolve over time. As a result, the conclusions drawn are based on data captured at a single point in time and may be subject to the influence of temporary conditions or other factors that were not measured or accounted for. Recognizing these limitations allows for better design of future research to achieve broader and more representative findings that will inform effective occupational health initiatives across the global maritime industry.

### 4.5. Future Research

Future research should aim to analyze data by gender to identify specific needs and tailor interventions accordingly. Moreover, while this study highlights several key stressors such as long working hours and high job demands, it does not delve deeply into the mechanisms through which these stressors affect mental health and well-being. Understanding these mechanisms is vital for developing targeted interventions that address not only the symptoms of stress but also its root causes.

Despite the significance of the obtained results, the effect sizes are rather small, indicating that the observed differences may have limited practical implications. Further research should explore longitudinal changes in stress levels and the potential influence of organizational culture and leadership styles. Recommendations for organizations should focus on holistic interventions, managerial support, and clear role definitions to reduce stress levels. Greater recognition of these aspects of organizational dynamics may assist maritime organizations in implementing comprehensive and sustainable interventions aimed at improving the well-being of seafarers, both at the individual and systemic levels.

## 5. Conclusions

This study aimed to identify the key occupational stressors among Lithuanian seafarers and to assess how these vary by years of service and job position. The findings confirmed that seafarers experience the most stress at work due to changes in work, relationships, peer support, and management support. Junior seafarers and seafarers with shorter years of service experience higher stress at work.

Seafarers with shorter years of service (0–11 years) experience more stress in role clarity (*p* = 0.004), demands (*p* = 0.005), relationships (*p* = 0.02), and work control (*p* = 0.035). Based on job position, junior seafarers experience significantly more stress in demands (*p* = 0.001) and role clarity (*p* = 0.009).

The study emphasizes the importance of putting more effort into enhancing organizational well-being to reduce occupational stress among seafarers.

## Figures and Tables

**Table 1:** Cronbach’s Alpha for the HSE Management Standards Indicator Tool and scale.

Scale and Item	α
**Demands** Item 3: Different groups at work demand things from me that are hard to combine Item 6: I have unachievable deadlines Item 9: I have to work very intensively Item 12: I have to neglect some tasks because I have too much to do Item 16: I am unable to take sufficient breaks Item 18: I am pressured to work long hours Item 20: I have to work very fast Item 22: I have unrealistic time pressures	0.760
**Managerial Support** Item 8: I am given supportive feedback on the work I do Item 23: I can rely on my line manager to help me out with a work problem Item 29: I can talk to my line manager about something that has upset or annoyed me about work Item 33: I am supported through emotionally demanding work Item 35: My line manager encourages me at work	0.820
**Peer support** Item 7: If work gets difficult, my colleagues will help me Item 24: I get help and support I need from colleagues Item 27: I receive the respect at work I deserve from my colleagues Item 31: My colleagues are willing to listen to my work-related problems	0.801
**Relationships** Item 5: I am subject to personal harassment in the form of unkind words or behavior Item 14: There is friction or anger between colleagues Item 21: I am subject to bullying at work Item 34: Relationships at work are strained	0.687
**Change at work** Item 26: I have sufficient opportunities to question managers about change at work Item 28: Staff are always consulted about change at work Item 32: When changes are made at work, I am clear how they will work out in practice	0.750
**Work control** Item 2: I can decide when to take a break Item 10: I have a say in my own work speed Item 15: I have a choice in deciding how I do my work Item 19: I have a choice in deciding what I do at work Item 25: I have some say over the way I work Item 30: My working time can be flexible	0.800
**Role clarity** Item 1: I am clear what is expected of me at work Item 4: I know how to go about getting my job done Item 11: I am clear what my duties and responsibilities are Item 13: I am clear about the goals and objectives for my department Item 17: I understand how my work fits into the overall aim of the organization	0.838
**Total**	0.766

**Table 2:** Statistics of the Kolmogorov-Smirnov criterion.

Subscale	Kolmogorov-Smirnov Statistic			Skewness	Kurtosis
	Statistic	df	*p*		
Demands	0.78	381	0.000	0.018	0.141
Managerial support	0.148	381	0.000	0.437	0.738
Peer support	0.164	381	0.000	0.814	1.310
Relationships	0.119	381	0.000	0.728	0.850
Change at work	0.178	381	0.000	0.786	1.254
Work control	0.120	381	0.000	−0.278	0.538
Role clarity	0.150	381	0.000	−1.295	2.306

**Table 3:** Indicators of stress subscales.

Variables of Subscales	Friedman Test		
	Mean	Mean Rank	*p* Value
**Demands**			
I have to work very intensively	3.47	6.51	χ^2^ = 720.424; dƒ = 7; *p* = 0.000
I have to work very fast	3.17	5.83	
I have to neglect some tasks because I have too much to do	2.57	4.25	
I have unrealistic time pressures	2.52	4.16	
Different groups at work demand things from me that are hard to combine	2.51	4.10	
I am unable to take sufficient breaks	2.46	4.01	
I am pressured to work long hours	2.37	3.83	
I have unachievable deadlines	2.14	3.22	
**Managerial support**			
I can rely on my line manager to help me out with a work problem	3.99	4.09	χ^2^ = 643.139; dƒ = 4; *p* = 0.000
I am given supportive feedback on the work I do	3.81	3.88	
My line manager encourages me at work	2.51	2.52	
I am supported through emotionally demanding work	2.44	2.47	
I can talk to my line manager about something that has upset or annoyed me about work	2.15	2.04	
**Peer support**			
If work gets difficult, my colleagues will help me	4.10	3.69	χ^2^ = 608.488; dƒ = 3; *p* = 0.000
My colleagues are willing to listen to my work-related problems	2.24	2.28	
I receive the respect at work that I deserve from my colleagues	2.05	2.08	
I get help and support I need from colleagues	1.98	1.96	
**Relationships**			
Relationships at work are strained	2.52	2.99	χ^2^ = 281.965; dƒ = 3; *p* = 0.000
There is friction or anger between colleagues	2.46	2.90	
I am subject to bullying at work	1.98	2.22	
I am subject to personal harassment in the form of unkind words or behavior	1.75	1.89	
**Change at work**			
When changes are made at work, I am clear how they will work out in practice	2.21	2.08	χ^2^ = 8.076; dƒ = 2; *p* = 0.018
Staff are always consulted about change at work	2.14	1.98	
I have sufficient opportunities to question managers about change at work	2.11	1.94	
**Work control**			
I can decide when to take a break	3.94	4.46	χ^2^ = 570.038; dƒ = 5; *p* = 0.000
I have some say over the way I work	3.86	4.37	
I have a choice in deciding how I do my work	3.69	4.02	
I have a choice in deciding what I do at work	3.17	3.11	
My working time can be flexible	2.77	2.82	
I have a say in my own work speed	2.34	2.22	
**Role clarity**			
I am clear what my duties and responsibilities are	4.59	3.39	χ^2^ = 127.852; dƒ = 4; *p* = 0.000
I am clear what is expected of me at work	4.53	3.20	
I am clear about the goals and objectives for my department	4.39	2.94	
I know how to go about getting my job done	4.41	2.87	
I understand how my work fits into the overall aim of the organization	4.22	2.61	

**Table 4:** Differences in HSE-MSIT subscales between groups (mean rank).

Variables	N	Demands	Managerial Support	Peer Support	Relationships	Change at Work	Work Control	Role Clarity
	(Total n = 385)	Mean Rank	Mean Rank	Mean Rank	Mean Rank	Mean Rank	Mean Rank	Mean Rank
**Years of service** 0–11 years 12 + years	205 180	178.05 210.03	209.83 180.66	194.25 191.57	180.69 207.02	193.84 190.99	180.39 204.10	177.99 210.10
Mann–Whitney U *p*		z = −2.821 *p* = 0.005	z = −2.077 *p* = 0.038	z = −0.241 *p* = 0.809	z = −2.333 *p* = 0.02	z = −0.257 *p* = 0.797	z = −2.113 *p* = 0.035	z = −2.859 *p* = 0.004
Effect size		−0.143	−0.106	-	−0.119	-	−0.108	−0.145
**Job position** Senior Junior	202 183	211.52 172.56	195.84 189.86	194.97 190.83	203.08 181.88	198.89 185.48	193.06 189.76	207.03 177.51
Mann–Whitney U *p*		z = −3.440 *p* = 0.001	z = −0.536 *p* = 0.592	z = −0.373 *p* = 0.709	z = −1.880 *p* = 0.06	z = −1.209 *p* = 0.227	z = −0.294 *p* = 0.768	z = −2.631 *p* = 0.009
Effect size		−0.175	-	-	-	-	-	−0.134

**Table 5:** Spearman’s correlations between years of service, job position, and HSE-MSIT subscales.

Variable/ Subscales	Years of Service	Job Position	Demands	Managerial Support	Peer Support	Relationships	Change at Work	Work Control	Role Clarity
**Years of service**									
**Job position**	−0.277 **								
**Demands**	0.144 **	−0.176 **							
**Managerial** **support**	−0.106 *	−0.027	0.133 **						
**Peer support**	−0.012	−0.019	0.378 **	0.314 **					
**Relationships**	0.119 *	−0.096	0.604 **	0.174 **	0.433 **				
**Change at work**	−0.013	−0.062	0.410 **	0.205 **	0.375 **	0.446 **			
**Work control**	0.108 *	−0.015	−0.209 **	−0.036	0.310 **	0.269 **	−0.309 **		
**Role** **clarity**	0.146 **	−0.134 **	−0.343 **	−0.115 *	−0.351 **	−0.458 **	−0.495 **	0.325 **	

** Correlation is significant at the 0.01 level. * Correlation is significant at the 0.05 level.

## Data Availability

The data are not publicly available due to confidentiality and privacy considerations. In addition, restrictions apply to the availability of these data due to our policy statement of sharing data. Data are available only on reasonable request.
